# Associations of Emotional Behavior with Nutritional Status and Lifestyle Habits among Schoolchildren Aged 5–10 Years in Sri Lanka

**DOI:** 10.3390/ijerph181910332

**Published:** 2021-09-30

**Authors:** Chisa Shinsugi, Deepa Gunasekara, Hidemi Takimoto

**Affiliations:** 1 Department of Nutritional Epidemiology and Shokuiku, National Institute of Health and Nutrition, National Institutes of Biomedical Innovation, Health and Nutrition, Tokyo 162-8636, Japan; shinsugi@nibiohn.go.jp; 2 Department of Biochemistry and Clinical Chemistry, Faculty of Medicine, University of Kelaniya, Ragama 11010, Sri Lanka; dcgune@gmail.com

**Keywords:** emotional and behavioral problems, nutritional status, lifestyle habits, schoolchildren, epidemiological study, Sri Lanka

## Abstract

We aimed to examine the relationships of lifestyle habits and nutritional status with emotional behavior among schoolchildren in Sri Lanka. Five hundred and eight schoolchildren (195 boys and 313 girls) aged 5–10 years were included. Emotional and behavioral problems were assessed using the Strengths and Difficulties Questionnaire. Nutritional status was used for body mass index-for-age *z*-score. Breakfast consumption, daily moderate- to vigorous-intensity physical activity (MVPA), wake-up time, and bedtime were considered lifestyle habits. The mean total difficulties score ± standard deviation was 12.0 ± 5.3, and the mean prosocial behavior score was 7.4 ± 1.9. In total, 89.2% children ate breakfast, and 41.3% engaged in at least 60 min of MVPA per day. After adjustment for confounding factors, multiple logistic regression analyses showed that breakfast skipping was associated with high scores on conduct problems (adjusted odds ratio (aOR) = 2.95, 95% confidence interval (CI): 1.50 to 5.77, *p* < 0.01) and that late bedtime was related to low prosocial behavior scores (aOR = 2.43, 95% CI: 1.17 to 5.03, *p* < 0.05). Our findings suggest that promoting regular lifestyle habits helps reduce psychological difficulties in schoolchildren. However, further research, including longitudinal studies, are required to identify the mechanism underlying this relationship.

## 1. Introduction

Emotional and behavioral problems among school-aged children are risk factors affecting healthy growth. Approximately 10–20% of children and adolescents worldwide have mental health problems that should be addressed to improve their well-being and productivity [1]. The World Health Organization (WHO) states that “Mental health is more than just the absence of mental disorders or disabilities. It is a state of well-being in which an individual realizes his or her own abilities, can cope with the normal stresses of life, can work productively and is able to make a contribution to his or her community” [2]. A previous study found a negative correlation between decreased academic performance and increased behavioral difficulties among Sri Lankan adolescents aged 10–13 years [3]. Some children may have difficulties in the school environment, where they learn social skills such as interpersonal communication and emotional control, in addition to academics. It is important to recognize those emotional and behavioral problems as early as possible to avoid truancy and delinquency. Although several complicating factors are associated with child mental health [1], current restrictions on daily life in particular, such as school closures because of the coronavirus disease 2019 (COVID-19) pandemic, may lead to psychological distress and increased mental health problems [4]. Moreover, there is concern regarding deteriorating mental health and increased behavioral problems among children and young people because of the pandemic [5]. Therefore, exploring the determinants of emotional behavior among community-dwelling schoolchildren in South Asia might help to identify corrective measures that are relatively easy to implement in daily life.

Unfavorable lifestyle behaviors, including skipping breakfast, low physical activity levels, and little or poor sleep, may be linked to mental health problems in children. Korean adolescents who skip breakfast have been shown to have an increased risk of stress and depressive mood [6], and children in the United Kingdom aged 11–14 years who met the recommended levels of physical activity (at least 1 h per day) had fewer emotional problems than did those with less physical activity after 1 year [7]. A systematic review found that shorter sleep duration was associated with poor emotional regulation in children and youth [8]. In Sri Lanka, nearly all children (99.1% in 2017) are enrolled in primary school [9]; therefore, teaching children healthy lifestyle habits and practices in primary school may be an effective way to prevent mental health problems. The Food-Based Dietary Guidelines for Sri Lankans recommend that school-aged children eat breakfast and participate in physical activity to promote a healthy lifestyle [10]. However, it remains unclear whether evidence for children in other countries can be generalized to children in South Asia. Children’s emotional and behavioral characteristics are often evaluated using the Strengths and Difficulties Questionnaire (SDQ) [11] or the Child Behavior Checklist (CBCL) [12]. The CBCL is a 113-item questionnaire focused on “problem behaviors” whereas the SDQ is a simple 25-item behavioral screening tool that assesses both “strengths and difficulties”. A previous study reported high correlations between scores on the SDQ and CBCL and showed that the SDQ had better detection of inattention and hyperactivity than did the CBCL [13]. Moreover, as the SDQ can be administered by non-clinical staff, such as children’s parents or teachers, and typically takes only 3–5 min to complete, the SDQ may be more appropriate for determining internalizing and externalizing problems in practical settings.

Physical growth status is another potential risk factor that may affect mental health. Australian children aged 4–7 years who were overweight or obese were found to be more likely to have high total scores reflecting difficulties compared with children who were normal weight [14]. In the Netherlands, young children aged 5–6 years who were underweight had lower scores for conduct problems than their counterparts who were normal weight; however, obese adolescents aged 13–14 years reported more peer problems and less prosocial behavior than normal-weight adolescents [15]. In Sri Lanka, severely malnourished adolescents in conflict-affected areas have higher psychosocial stress scores than adolescents with healthy weight [16]; however, this result was found under special circumstances and may not be able to be replicated in general populations of community-dwelling schoolchildren. Furthermore, the association between emotional behavior and undernutrition among children in primary school should be evaluated in circumstances where undernutrition is highly prevalent. The 2015 Annual Health Bulletin indicated that the proportion of wasting and stunting among primary schoolchildren aged 5–6 years was 20.3% and 8.7%, respectively [17]. Therefore, in the present study, we aimed to examine the relationships of emotional behavior with both nutritional status and lifestyle factors among primary schoolchildren in Sri Lanka.

## 2. Material and Methods

### 2.1. Study Participants

**Figure 1:**
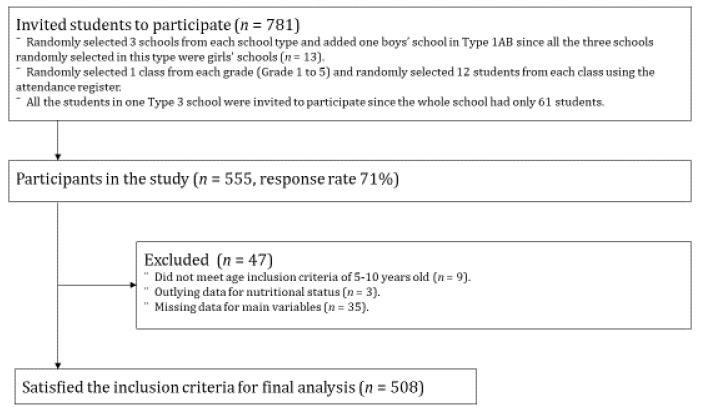
Participant flow chart in the sample for analysis.

The data in this study were obtained from a school-based cross-sectional study conducted in September 2017 in Sri Lanka. All primary schools were categorized into four types, in accordance with the educational system in Sri Lanka: Type 1AB (13-year education in the arts and sciences), Type 1C (13-year education in the arts), Type 2 (11-year education), and Type 3 (5-year education). The study involved 13 primary schools from four school types in Gampaha District, Sri Lanka, and the participants were randomly selected. Details of the sampling methods have been described previously [18]. After obtaining written informed consent from the children’s parents or guardians to enroll them in the study, we collected anthropometric measurements of the children and administered a self-administered questionnaire that was completed by a parent or guardian of each participating child. Of the 555 responses (response rate: 71%), 9 were excluded because the child was not in the appropriate age range (5–10 years old), 3 were excluded for being outliers in terms of nutritional status, and 35 were excluded for missing data for the main variables (*n* = 12 for SDQ score, *n* = 18 for lifestyle habits, and *n* = 5 for number of siblings). Ultimately, the sample size for analysis included 508 children. A participant flow chart is shown in Figure 1. The study protocol of this study was approved by the Ethical Review Committee of the University of Kelaniya in Sri Lanka; the Institutional Review Board of the National Institutes of Biomedical Innovation, Health and Nutrition in Japan (No. 150, June 2017); and the Department of Education, Western Province, Sri Lanka.

### 2.2. Dependent Variables

#### Psychopathology Data

Emotional and behavioral problems were assessed using the Sinhala version of the SDQ [11,19]. The SDQ comprises 25 items that are categorized into five groups: emotional symptoms, conduct problems, hyperactivity–inattention, peer problems, and prosocial behavior. The total difficulties score (TDS) is generated from all categories except prosocial behavior. Each item is scored on a three-point scale (not true, somewhat true, certainly true), and each subscale has five items and a score range of 0–10. The TDS comprises 20 items with an overall score range of 0–40. Higher scores on prosocial behavior reflect strengths (favorable), whereas higher scores on the other four subscales and the TDS reflect difficulties (problematic). In this study, the Cronbach’s alpha values for the TDS and prosocial behavior scores were 0.75 and 0.70, respectively, which indicated reliable internal consistency. With reference to a previous study [20], the abnormal ranges were defined as follows: emotion (7–10), conduct (5–10), hyperactivity (7–10), peer problems (5–10), prosocial (0–5), and TDS (19–40).

### 2.3. Independent Variables

#### 2.3.1. Anthropometric Measurements

Children’s height was measured to the nearest 0.1 cm using a portable stadiometer (Seca, Hangzhou, China) with the child standing barefoot. Weight was measured to the nearest 0.1 kg using a digital scale (Seca, Hangzhou, China) with the child wearing light clothing. Body mass index was calculated as weight divided by the square of height. The body mass index-for-age *z*-score (BAZ) was determined using the WHO growth standards [21]. Thinness was defined as BAZ < −2. Overweight and obesity were classified as BAZ > 1.

#### 2.3.2. Lifestyle Habits

Breakfast skipping, physical activity, wake-up time, and bedtime were considered as lifestyle habits. Breakfast skipping was assessed according to whether the child usually consumed breakfast in accordance with the Food-Based Dietary Guidelines for Sri Lankan children aged 5–10 years [10]. The frequency and duration of participants’ physical activity during a typical week were reported by their guardians. We asked about three types of physical activity (vigorous-, moderate-, and light-intensity activities), with reference to the Global Physical Activity Questionnaire [22] and the Global School-based Student Health Survey [23]. The WHO recommends that children and youth aged 5–17 years engage in at least 60 min of moderate- to vigorous-intensity physical activity (MVPA) daily; therefore, we calculated the total amount of MVPA. We asked the children’s parents/guardians about their child’s wake-up time and bedtime on a normal day. We established three categories for wake-up time: <6:00 am, 6:00 am to 6:29 am, and 6:30 am to 8:29 am. We categorized bedtime as 7:00 pm to 8:59 pm, 9:00 pm to 9:59 pm, and 10:00 pm. to 11:59 pm. We defined an early wake-up time as waking up before 6:00 am and a late bedtime as going to bed after 10:00 pm.

### 2.4. Covariates

Sex, age, number of siblings (0–4), and school type (1AB, 1C, 2, or 3) were included as covariates.

### 2.5. Statistical Analysis

Using multiple logistic regression models, we examined the associations of emotional and behavioral problems (each SDQ subgroup) with both nutritional status and lifestyle habits (breakfast intake, MVPA, wake-up time, and bedtime) after adjusting for sex, age, number of siblings, and school type. The statistical significance level was set at *p* < 0.05. The statistical analyses were performed using Stata version 15.1 (StataCorp LLC, College Station, TX, USA).

## 3. Results

The characteristics of the included participants are shown in Table 1. The mean TDS score ± standard deviation was 12.0 ± 5.3, and the mean score for prosocial behavior was 7.4 ± 1.9. In total, 13.2% of children had conduct problems and 18.3% had prosocial behavior problems. As for lifestyle habits, most children ate breakfast (89.2%), and nearly half engaged in at least 60 min of MVPA per day (41.3%). Approximately one in three children woke up early (before 6 am) (36.8%), and only 11.4% of children went to bed late (after 10 pm).

Table 2 shows the results of the multiple logistic regression analyses of the associations of emotional and behavioral problems with both nutritional status and lifestyle habits. After adjustment for confounding factors, breakfast skipping was associated with high scores for conduct problems (adjusted odds ratio (aOR) = 2.95, 95% confidence interval (CI): 1.50 to 5.77, *p* < 0.01), whereas late bedtime was related to low scores for prosocial behavior (aOR = 2.43, 95% CI: 1.17 to 5.03, *p* < 0.05).

## 4. Discussion

In this cross-sectional study, we examined whether nutritional status or lifestyle habits, including breakfast intake, physical activity, wake-up time, and bedtime, are associated with psychological behavior among schoolchildren aged 5–10 years in Sri Lanka. We found that unfavorable lifestyle habits such as breakfast skipping and late bedtime were related to emotional and behavioral problem factors (i.e., conduct problems and prosocial behavior problems). No associations between nutritional status and emotional behavior were observed.

The mean TDS in this study (12.01) was slightly higher than that reported in a previous study (10.33) among adolescents aged 12–16 years across Sri Lanka [20]. Possible reasons for the disparity include differences in the study areas, the age of participants, and the types of respondents providing the data (i.e., parents or self-reporting). The target area of this study in Gampaha District was a relatively urban area, and the children may be more likely than children living in more rural areas to experience intensive stress in their daily lives, such as pressure to achieve high academic performance. Additionally, primary school-aged children may be more prone to emotional problems than adolescents because children in primary school are still in the process of developing mentally and face more challenges in controlling their psychological states. The phenomenon of SDQ scores tending to be lower for adolescents than for school-aged children is consistent with the results of a previous study conducted in Japan [24]. Additionally, the same Japanese study showed the mean SDQ scores as assessed by parents were higher than those assessed by teachers. There are no self-report SDQ data for children aged 10 years or younger, and it is inappropriate to compare parental assessment of younger children with self-reports from older children. Thus, the differences in respondent type may have contributed to the differences in scores between these two studies in Sri Lanka.

In the present study, schoolchildren who were breakfast skippers were more likely than those who were not to have conduct problems, after adjustment for confounding factors. This finding is consistent with those of a previous study among Iranian children and adolescents showing that students who skipped breakfast were more likely than those who did not to have mental health difficulties, such as depression and anxiety, and to exhibit violent behavior, including bullying and physical fighting [25]. Moreover, previous studies have reported associations between skipping breakfast and low self-esteem [26] and spending more time using social networking sites [27]. In Japan, *shokuiku* (food and nutrition education) lectures that include information on the importance of breakfast are provided to schoolchildren and their families throughout the country [28]. The introduction of school meal programs along with nutritional education as part of the school curriculum may be key interventions in primary schools to improve children’s mental health status.

In this study, children with late bedtimes were found to be more likely than others to have prosocial behavior problems. A previous study showed that Chinese preschool-aged children with longer screen times (≥2 h per day) and shorter sleep duration (<9.15 h/day) had an increased risk of prosocial problems [29]. Moreover, in Australia, it is recommended that children aged 5–10 sleep at least 9–10 h per day; additionally, shorter sleep duration (<10 h), bedtime latency on non-school days, and bedtime variability on school days have been linked to behavioral problems [30]. This phenomenon might be because children with late bedtimes tend to be sleep-deprived during the day and may be less considerate of their friends and others. Further studies are required to examine these mechanisms and to explore effective support to promote regular sleep habits and address prosocial behavior problems in children.

This study has several limitations. First, because this was a cross-sectional study, conclusions regarding causation cannot be drawn from the results. Second, we used the international definition of nutritional status based on the WHO growth standards because of the absence of standards specific to Sri Lankan children. Third, we collected physical activity data using a questionnaire rather than using direct measurement methods. However, we referred to the Global Physical Activity Questionnaire, which has been used in more than 100 countries globally for WHO surveillance. Fourth, we could not eliminate the impact of potential confounding factors, such as residual and unmeasured variables. Fifth, the sample size in this study may not have been sufficiently large. Although these limitations must be considered when interpreting the results, this study revealed important information regarding the associations of emotional behavior with both nutritional status and lifestyle habits among primary schoolchildren in Sri Lanka. Unfavorable lifestyle habits are more concerning during the COVID-19 pandemic, and there is an increasing need to address these lifestyle issues to improve the mental health of schoolchildren, not only in high-income countries but also in Sri Lanka and other low- and middle-income countries.

## 5. Conclusions

The present study’s findings showed that, among Sri Lankan primary schoolchildren, unfavorable lifestyle habits such as breakfast skipping and late bedtime were related to psychological difficulties including conduct problems and lower levels of prosocial behavior. Our findings suggest that lifestyle habits may play an important role in reducing emotional and behavioral problems among children in Sri Lanka. To enhance healthy growth in this vulnerable population, further studies are needed to help policymakers develop strategies and allocate human and financial resources to expanding mental health support and education on healthy lifestyle habits in school environments.

## Figures and Tables

**Table 1:** Characteristics of schoolchildren (5–10 years old) in Sri Lanka (*n* = 508).

Variable	Total		Variable	Total	
	*N* or Mean	% or SD		*N* or Mean	% or SD
**Sex**			**Emotional behavior (SDQ, mean, SD)**		
Boy	195	38.4	Total difficulties score (TDS, 0–40)	12.01	5.26
Girl	313	61.6	Conduct problems (0–10)	2.53	1.81
**Age (years)**			Hyperactivity–inattention (0–10)	4.00	2.16
5	39	7.7	Emotional symptoms (0–10)	2.24	1.90
6	112	22.1	Peer problems (0–10)	3.23	1.47
7	94	18.5	Prosocial behavior (0–10)	7.39	1.94
8	106	20.9	**Abnormal (SDQ, *N*, %)**		
9	99	19.5	TDS (19–40)	57	11.2
10	58	11.4	Conduct problems (5–10)	67	13.2
**Number of siblings**			Hyperactivity–inattention (7–10)	68	13.4
0	96	18.9	Emotional symptoms (7–10)	20	3.9
1	242	47.6	Peer problems (5–10)	89	17.5
2	141	27.8	Prosocial behavior (0–5)	93	18.3
3	28	5.5	**Nutritional Status**		
4	1	0.2	BAZ, (mean, SD)	−0.80	1.48
**School type**			BAZ (%)		
1AB	157	30.9	Thin (<–2)	99	19.5
1C	85	16.7	Normal weight (–2 to +1)	342	67.3
2	128	25.2	Overweight or obese (>+1)	67	13.2
3	138	27.2	**Lifestyle habits**		
			Breakfast intake		
			No	55	10.8
			Yes	453	89.2
			MVPA (per day)		
			<60 min	298	58.7
			≥60 min	210	41.3
			Wake–up time		
			<6:00 am	187	36.8
			6:00 am–6:29 am	278	54.7
			6:30 am–8:29 am	43	8.5
			Bedtime		
			7:00 pm–8:59 pm	239	47.1
			9:00 pm–9:59 pm	211	41.5
			10:00 pm–11:59 pm	58	11.4

SD, standard deviation; SDQ, Strengths and Difficulties Questionnaire; BAZ, body mass index-for-age *z*-score; MVPA, moderate- to vigorous-intensity physical activity. Total difficulties score (TDS) = conduct problems + hyperactivity-inattention + emotional symptoms + peer problems.

**Table 2:** Associations of emotional and behavioral problems with nutritional status and lifestyle habits among schoolchildren in Sri Lanka (*n* = 508).

**Variables**	**High TDS** **(19** **–40)**			**Conduct Problems** **(5–10)**			**Hyperactivity-Inattention (7–10)**		
	**aOR**	**95% CI**	***p***	**aOR**	**95% CI**	***p***	**aOR**	**95% CI**	***p***
**Nutritional status**									
Thin (BAZ < −2)	1.26	(0.64–2.49)		1.26	(0.65–2.44)		0.90	(0.46–1.74)	
Normal weight (−2 to +1)	1.00			1.00			1.00		
Overweight or obese (BAZ > +1)	0.60	(0.22–1.65)		1.25	(0.56–2.82)		0.48	(0.18–1.28)	
**Lifestyle habits**									
Breakfast intake—no (1), yes (0)	1.54	(0.69–3.42)		2.95	(1.50–5.77)	**	1.76	(0.84–3.70)	
MVPA (per day)—<60 (1), ≥60 (0)	1.02	(0.57–1.83)		0.85	(0.49–1.48)		0.79	(0.46–1.36)	
Wake-up time									
<6:00 am	1.00			1.00			1.00		
6:00 am–6:29 am	0.91	(0.50–1.68)		1.00	(0.55–1.79)		0.95	(0.53–1.69)	
6:30 am–8:29 am	0.77	(0.24–2.47)		1.50	(0.57–3.97)		1.10	(0.42–2.87)	
Bedtime									
7:00 pm–8:59 pm	1.00			1.00			1.00		
9:00 pm–9:59 pm	0.84	(0.45–1.56)		0.88	(0.50–1.57)		0.81	(0.45–1.44)	
10:00 pm–11:59 pm	1.51	(0.64–3.54)		1.20	(0.50–2.88)		1.38	(0.62–3.11)	
**Variables**	**Emotional Symptoms** **(7–10)**			**Peer Problems** **(5–10)**			**Prosocial Behavior Problems (0–5)**		
	**aOR**	**95% CI**	***p***	**aOR**	**95% CI**	***p***	**aOR**	**95% CI**	***p***
**Nutritional status**									
Thin (BAZ < −2)	2.04	(0.70–5.94)		0.78	(0.42–1.43)		1.06	(0.59–1.92)	
Normal weight (−2 to +1)	1.00			1.00			1.00		
Overweight or obese (BAZ > +1)	1.50	(0.37–6.00)		0.69	(0.32–1.49)		0.63	(0.28–1.41)	
**Lifestyle habits**									
Breakfast intake—no (1), yes (0)	0.38	(0.05–3.00)		1.69	(0.86–3.32)		1.64	(0.82–3.29)	
MVPA (per day)—<60 (1), ≥60 (0)	0.81	(0.31–2.12)		1.01	(0.62–1.65)		1.20	(0.73–1.97)	
Wake-up time									
<6:00 am	1.00			1.00			1.00		
6:00 am–6:29 am	0.91	(0.33–2.50)		1.46	(0.88–2.44)		1.38	(0.80–2.37)	
6:30 am–8:29 am	1.53	(0.28–8.29)		0.58	(0.19–1.79)		1.75	(0.75–4.07)	
Bedtime									
7:00 pm–8:59 pm	1.00			1.00			1.00		
9:00 pm–9:59 pm	2.07	(0.72–5.96)		0.80	(0.48–1.32)		1.21	(0.72–2.04)	
10:00 pm–11:59 pm	2.91	(0.75–11.30)		1.12	(0.53–2.38)		2.43	(1.17–5.03)	*

aOR, adjusted odds ratio; CI, confidence interval; BAZ, body mass index-for-age *z*-score; MVPA, moderate- to vigorous-intensity physical activity; SDQ, Strengths and Difficulties Questionnaire; TDS, total difficulties score. ** *p* < 0.01, * *p* < 0.05. The associations of SDQ with both nutritional status and lifestyle factors were examined after adjustment for sex, age, number of siblings, and school type. TDS = conduct problems + hyperactivity-inattention + emotional symptoms + peer problems.

## Data Availability

Interested and qualified researchers who meet the criteria for access to confidential data can request data access from Faculty of Medicine, the University of Kelaniya.
